# A 41-Gene Pair Signature for Predicting the Pathological Response of Locally Advanced Rectal Cancer to Neoadjuvant Chemoradiation

**DOI:** 10.3389/fmed.2021.744295

**Published:** 2021-09-14

**Authors:** Zhengfa Xue, Shuxin Yang, Yun Luo, Hao Cai, Ming He, Youping Ding, Lei Lei, Wei Peng, Guini Hong, You Guo

**Affiliations:** ^1^School of Information Engineering, Jiangxi University of Science and Technology, Ganzhou, China; ^2^Medical Big Data and Bioinformatics Research Centre, First Affiliated Hospital of Gannan Medical University, Ganzhou, China; ^3^School of Medical Information Engineering, Gannan Medical University, Ganzhou, China

**Keywords:** rectal cancer, neoadjuvant chemoradiation, relative expression orderings, signature, survival

## Abstract

**Background and Purpose:** Pathological response status is a standard reference for the early evaluation of the effect of neoadjuvant chemoradiation (nCRT) on locally advanced rectal cancer (LARC) patients. Various patients respond differently to nCRT, but identifying the pathological response of LARC to nCRT remains a challenge. Therefore, we aimed to identify a signature that can predict the response of LARC to nCRT.

**Material and Methods:** The gene expression profiles of 111 LARC patients receiving fluorouracil-based nCRT were used to obtain gene pairs with within-sample relative expression orderings related to pathological response. These reversal gene pairs were ranked according to the mean decrease Gini index provided by the random forest algorithm to obtain the signature. This signature was verified in two public cohorts of 46 and 42 samples, and a cohort of 33 samples measured at our laboratory. In addition, the signature was used to predict disease-free survival benefits in a series of colorectal cancer datasets.

**Results:** A 41-gene pair signature (41-GPS) was identified in the training cohort with an accuracy of 84.68% and an area under the receiver operating characteristic curve (AUC) of 0.94. In the two public test cohorts, the accuracy was 93.37 and 73.81%, with AUCs of 0.97 and 0.86, respectively. In our dataset, the AUC was 0.80. The results of the survival analysis show that 41-GPS plays an effective role in identifying patients who will respond to nCRT and have a better prognosis.

**Conclusion:** The signature consisting of 41 gene pairs can robustly predict the clinical pathological response of LARC patients to nCRT.

## Introduction

Neoadjuvant chemoradiation (nCRT) followed by surgery is an effective treatment for locally advanced rectal cancer (LARC) (1). This treatment is considered a safe and effective strategy for locally advanced colon cancer (2). The pathological response to nCRT determines surgical plan and post-operative quality of life of LARC patients (3). Pathological responders can benefit from nCRT, whereas non-pathological responders can choose not to undergo nCRT to avoid the risk and pain associated with this type of treatment. Nevertheless, among patients with LARC, the proportion of patients with pathological complete response and partial pathological response is only ~10–25% and 40%, respectively (4). Therefore, predicting the nCRT response before treatment may substantially improve the choice of patients for pre-operative chemotherapy.

Several prediction models based on tumor tissue expression profiles have shown high accuracy on their respective datasets (5–12), but the high variability and batch effects make it difficult to apply these predictive models to independent data (13). In addition, the standardization process of adjusting batch effects in gene expression profiling requires the collection of a certain number of samples, which delays the subsequent treatment of patients in clinical practice (14). The accuracy of model prediction will also be affected by sample normalization (15). Our previous evidence showed that the within-sample relative expression orderings (REOs) of gene pairs (16) can robustly resist batch effects, and we have successfully screened gene pairs with marked differences between responders and non-responders of LARC (17). However, the selection of gene pairs is still a problem worthy of discussion, as the accuracy varies among predictive signatures composed of different gene pairs. The random forest algorithm (18) can effectively analyze high-dimensional data (the number of variables is more than a hundred times the number of observations), and at the same time provide a variable importance measure (VIM), which makes random forest especially suitable for the study of expression profile to identify potential biomarkers (19). Compared to our previous research, we can measure how important a gene is by the size of the VIM value, rather than saying that the gene is useful or useless. This can prevent the screening of genes that have a classification effect.

This study aimed to screen gene pairs using the VIM values of random forest, thereby exploring the gene pair signature (GPS) in predicting the response of LARC to nCRT.

## Materials and Methods

### Data and Pre-processing

A total of 232 rectal cancer patients were included in this study. They were from three public cohorts of LARC, namely GSE87211 (20), GSE35452 (21), and GSE45404 (22), and a cohort measured at our laboratory. The surgical plan for all patients was neoadjuvant fluorouracil (5-FU)-based chemoradiation combined with 50.4 Gy radiotherapy.

In GSE87211, 363 samples were measured using the Agilent-026652 whole human genome microarray 4 × 44K v2 (GPL13497) platform, including 203 patients with rectal cancer. We screened 111 patients who received nCRT and provided pathological response information. The pathological response of these patients was determined according to the American Joint Committee on Cancer tumor regression grade (TRG) (23).

The validation cohorts GSE35452 and GSE45404 were measured using the Affymetrix Human Genome U133 Plus 2.0 Array (GPL570) platform. These cohorts comprised 46 and 42 rectal cancer patients who received nCRT, respectively. The response status of these patients was assessed using the Mandard TRG system. Although the classification standards of the Mandard and AJCC TRG systems are slightly different, the difference between them is negligible (23). The last validation cohort with 33 LARC patients was from Fujian Medical University Union Hospital (17).

Because the pathological response status has a positive effect on disease-free survival (DFS) (24), the 41-GPS should be used to predict the survival benefit of 5-FU-based nCRT. First, we used the signature to evaluate the survival benefit of 105 patients with rectal cancer treated with 5-FU-based nCRT in the GSE87211 cohort. To further verify our signature, we used the 41-GPS to predict the survival benefits of 285 patients with colorectal cancer. The information of these patients was in reference (17). Because rectal and colon cancers are almost genetically indistinguishable (25), we expected that the 41-GPS would also be applicable to patients with colon cancer. We also collected 158 The Cancer Genome Atlas Rectum Adenocarcinoma (TCGA-READ) to predict DFS benefits.

In addition, nine colorectal cancer (CRC) cell lines in GSE20298 (26) were used in this study, including Caco-2, LS513, LS1034, SW403, SW480, SW620, SW837, SW1116, and SW1463. We used these to evaluate the resistance of the cell lines, which is measured by the survival rate of tumor cells under 3 μM/L 5-FU and 2 Gy X-ray radiation exposure.

TCGA cohorts were obtained from the Genomic Data Commons Data Portal, whereas others were obtained from the Gene Expression Omnibus repository (27). All data are standardized by the Robust Multi-Array Average algorithm (28) and are shown in Table 1. Data normalization of the inter-sample was not performed. When multiple probes were mapped to a gene symbol, the arithmetic mean of the probe values was used (on the log 2 scale).

**Table 1:** Datasets analyzed in this study.

**GEO ID**	**Describe**	**Platform**	**Sample size**	**Tissue sample type**
GSE87211	Expression profile	GPL13497	111	Rectal cancer
GSE35452	Expression profile	GPL570	46	Rectal cancer
GSE45404	Expression profile	GPL570	42	Rectal cancer
GSE39582	Expression profile	GPL570	200	Colorectal cancer
GSE14333	Expression profile	GPL570	85	Colorectal cancer
TCGA	Expression profile	-	158	READ
GSE20298	Cell lines	GPL4133	-	Colorectal cancer
Our dataset	Expression profile	GPL15207	33	Rectal cancer

### Procedure of Discovering Signature

**Figure 1:**
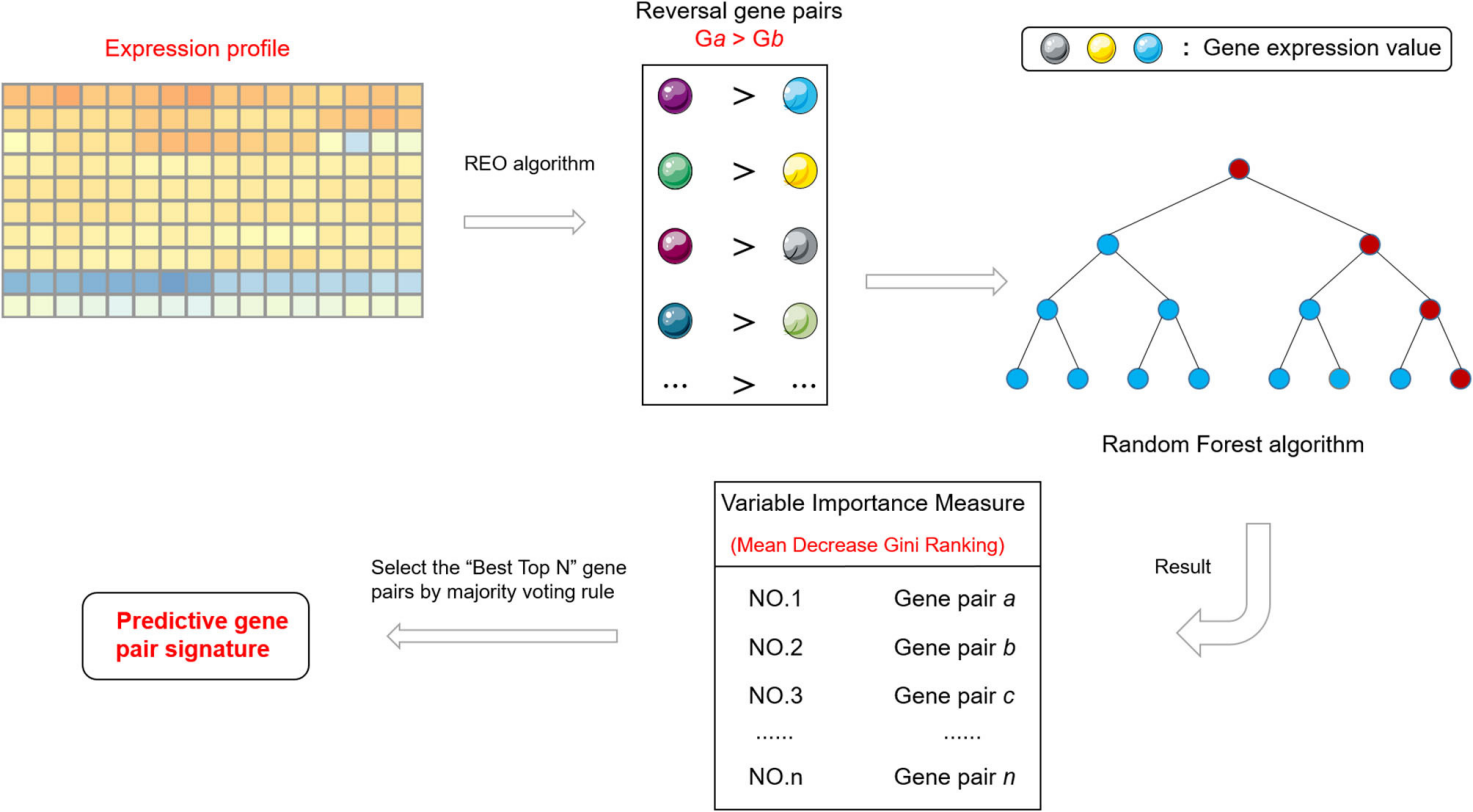
Flowchart of signature discovery process and validation in this study.

The signature discovery process is illustrated in Figure 1. First, according to the Tumor Regression Classification (TRG) of the American Joint Cancer Council of the United States, patients in the training group were divided into two groups, namely the response and the non-response group. Then the REO algorithm was used to obtain the reversal gene pairs of the training set (Binomial test, *p* < 0.05). The reversal gene pair includes two genes, gene *a* and *b*, and the relationship was recorded as *G*_*a*_ > *G*_*b*_ or *G*_*a*_<*G*_*b*_, where *G*_*a*_ and *G*_*b*_ represent expression values. The reversal gene pairs were then inputted into the random forest algorithm to obtain the VIM ranking according to the mean decrease Gini (MDG) index. Finally, the best top-N gene pairs were selected as the signature, where best top-N is the top N gene pairs having the highest classification accuracy with the majority voting rule in the training dataset.

### Statistical Analysis

The open-source statistical analysis software R4.0.2, with R packages *randomForest, rms, limma*, and *survival*, was used for statistical analysis. The reversal gene pair obtained using the REO algorithm was evaluated using a Binomial test (*p* < 0.05). The online software DAVID Bioinformatics Resources 6.8 was used for gene ontology (GO) enrichment analysis. Term with a *P*-value <0.05 was considered statistically significant. The R package *limma* is used to identify differentially expressed genes. The accuracy of the signature is defined as the ratio of the correctly predicted samples to all samples in the cohort of responders and non-responders, and the 95% confidence interval was calculated by the Clopper-Pearson method (29). The parameters of the best random forest model in the seed 20200914 were “best mtry” = 88 and “ntree” = 2,500. Receiver operating characteristic (ROC) curve and area under the curve (AUC) were used to assess the predictive ability of the signature. The Kaplan–Meier method and log-rank test were used to evaluate the survival analysis of the training dataset and TCGA dataset. DFS was defined as the time from surgery to relapse or the date of final recording (24). In all statistical analyses, P <0.05 was considered statistically significant.

## Results

### Establishing the 41 Gene Pair Signature for Response Prediction in LARC

**Figure 2:**
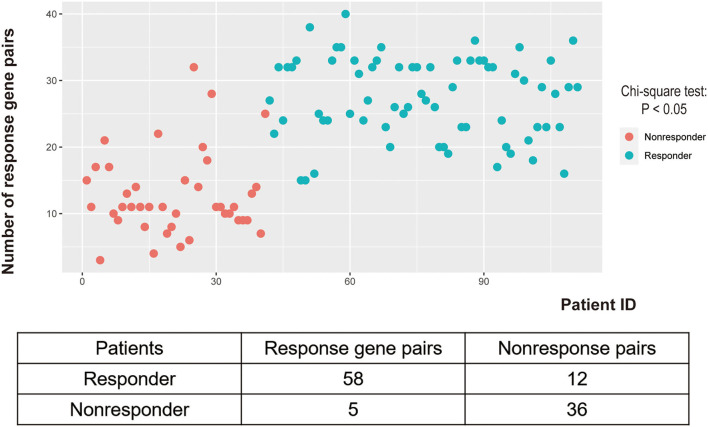
Distribution of response gene pairs in patients of GSE87211 under 41-GPS prediction.

The discovery workflow is shown in Figure 1. Using GSE87211 as training data and REO algorithm, we identified 13,388 reversal gene pairs that were significantly related to the pathological response status of LARC patients receiving nCRT (Binomial test, *p* < 0.05). We then introduced all reversal gene pairs into the random forest model to obtain the VIM value. The gene pairs were sorted in order of decreasing importance of MDG. We selected 41 gene pairs using the majority voting rule to establish the signature (referred to as 41-GPS; Table 2) that achieved the highest classification accuracy. In the training dataset, 58 of 70 responders (sensitivity = 82.86%) and 36 of 41 non-responders (specificity = 87.80%) were correctly classified using the 41-GPS signature (Figure 2). The overall accuracy was 84.68% (95% CI, 81.27–88.10%), and the AUC was 0.946 (95% CI, 0.901–0.990) (**Figure 4A**).

**Table 2:** The composition of 41-GPS.

**Gene pairs**	**Gene pairs**	**Gene pairs**
(G_***a***_ > G_b_^*^)	**(*G_***a***_ > G_***b***_*)**	**(*G_***a***_ > G_***b***_*)**
*1. ABCC5 > FUT8*	*15. USP19 > HENMT1*	*29. TUBB2A > NENF*
*2. EFNA1 > CDKN3*	*16. ECSIT > COA8*	*30. PRKCZ > TIMM9*
*3. AGRN > UBE2L6*	*17. AMBR A1> SOCS4*	*31. BPNT1 > TMEM39B*
*4. SLC17A9 > SUPT7L*	*18. GLYCTK > ERP27*	*32. ASPHD1 > THAP1*
*5. EPS8L2 > PARN*	*19. EBF1 > ARHGEF39*	*33. SLC17A9 > APOL2*
*6. ITGA3 > MIS18BP1*	*20. MIER3 > GSTT1*	*34. DHFR2 > LPCAT2*
*7. ZP1 > LTA*	*21. MICAL1 > ZNF92*	*35. KMT2B > AJUBA*
*8. CRY2 > RHOBTB1*	*22. ZNF460 > WDR66*	*36. LHFPL2 > COA8*
*9. SESTD1 > CCDC25*	*23. PRELID2 > PRMT5*	*37. SFI1 > DCP1B*
*10. GOT1 > WASHC3*	*24. DCAF1 > TBC1D5*	*38. DCTPP1 > ENY2*
*11. NPHP4 > WDCP*	*25. PRRC1 > PPP1R2*	*39. HAGHL > KLHDC9*
*12. DHFR2 > NME7*	*26. STRIP1 > KIN*	*40. CRTC1 > GSTT1*
*13. EHBP1L1 > LPCAT2*	*27. GLYAT > DKK4*	*41. ASPHD1 > SMUG1*
*14. IWS1 > TSEN15*	*28. FZD9 > CXCL11*	

* *G_a_ > G_b_ represents the response to neoadjuvant chemoradiation in locally advanced rectal cancer*.

**Figure 3:**
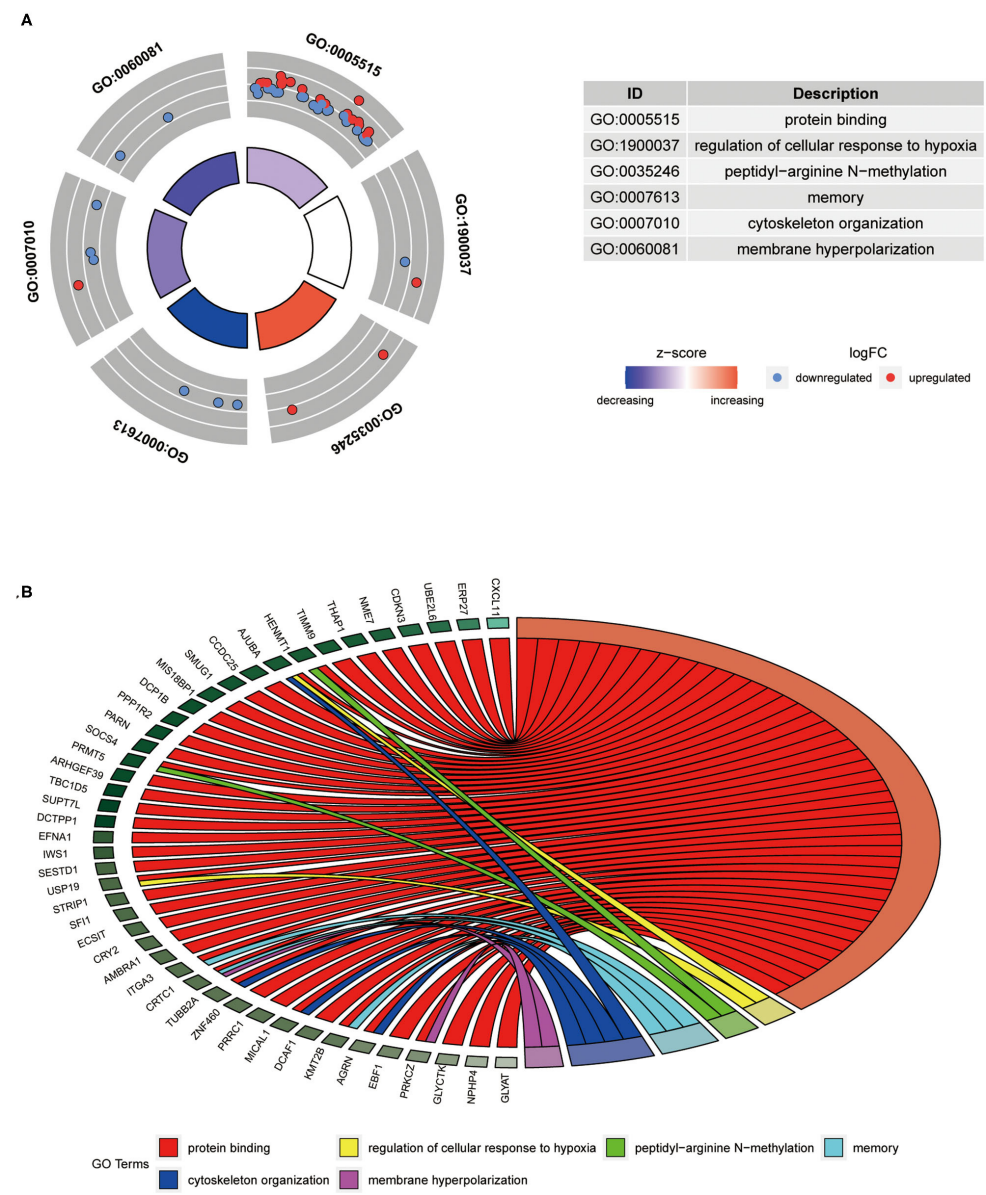
Composition and differentially expressed genes of 41-GPS. **(A)** Gene enrichment distribution map. The picture shows the six most significant enrichment pathways (p<0.05). Dots represent the gene symbol, red denotes upregulated gene, and blue denotes downregulated genes. The color of the enrichment pathway indicates the size of the z-score. **(B)** The specific distribution of gene enrichment. Different colors represent different access pathways.

The 41-GPS contains 76 genes, nearly half of which (37 of 76) were differentially expressed between the responder and non-responder cohorts (*t*-test, *p* < 0.05) (Figure 3A). The overlapping genes in the signature were ASPHD1, COA8, DHFR2, GSTT1, LPCAT2, and SLC17A9. GO enrichment analysis revealed that 57.89% of the signature genes were enriched in the protein binding of molecular function pathway (*p* < 0.05; Figure 3B).

### The 41-GPS Is a Strong Predictor in Independent Cohorts of LARC

**Figure 4:**
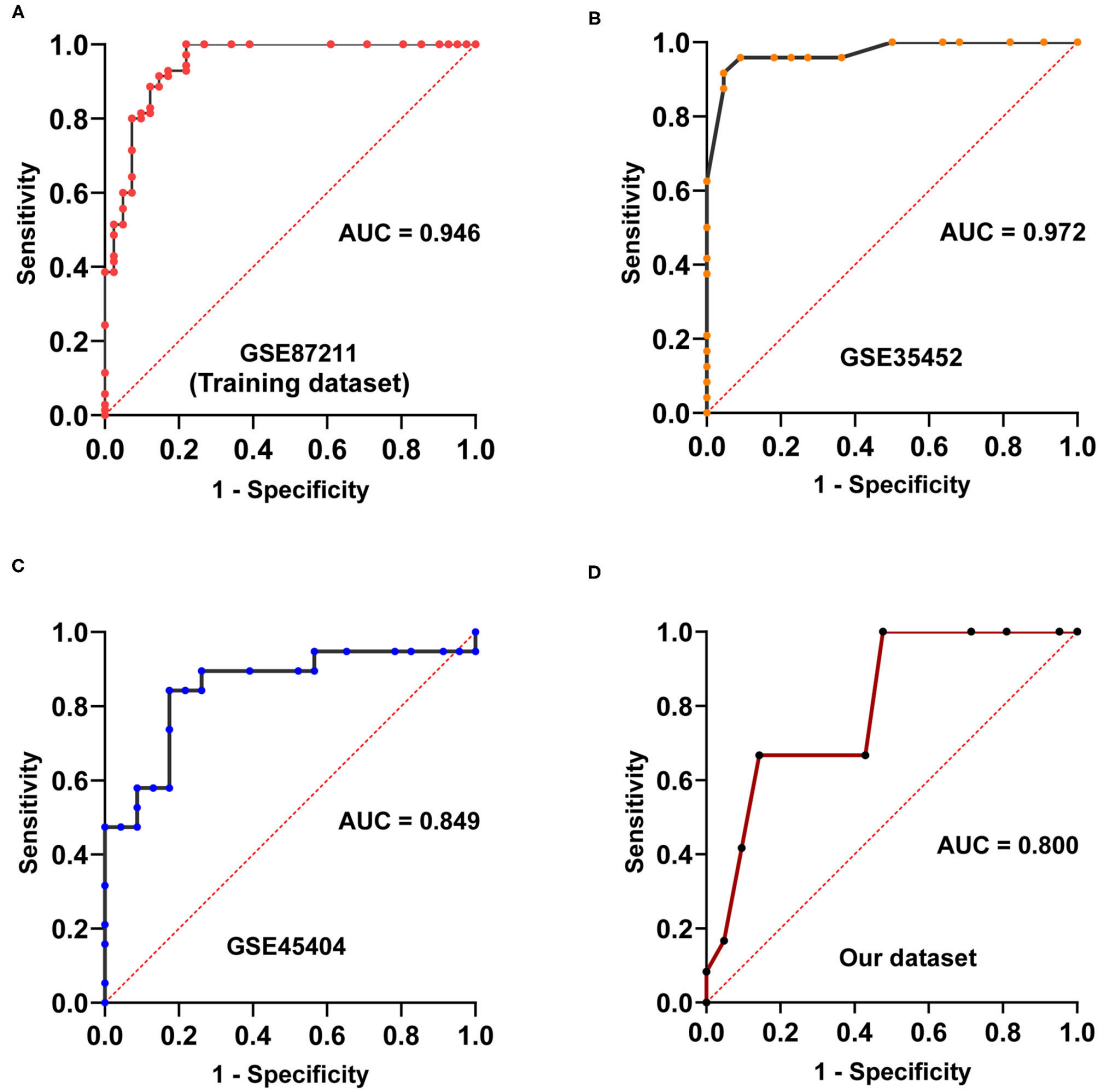
Area under receiver characteristic operating curves of four cohorts. **(A)** Area under receiver characteristic operating curves of GSE87211. **(B)** Area under receiver characteristic operating curves of GSE35452. **(C)** Area under receiver characteristic operating curves of GSE45404. **(D)** Area under receiver characteristic operating curves of the dataset measured in our laboratory.

In the first verification cohort (GSE35452), the signature predicted nCRT responders with a sensitivity and specificity index of 95.83% (23/24) and 90.90% (20/22), respectively, and an accuracy of 93.37% (95% CI, 89.84–97.12%). In the second validation cohort (GSE45404), the sensitivity and specificity of the prediction of nCRT responders were 89.47% (17/19) and 60.87% (14/23), respectively, and the accuracy was 73.81% (95% CI, 67.03–80.59%). The AUC of the two validation cohorts was 0.972 (95% CI, 0.929–1) and 0.849 (95% CI, 0.722–0.976) in that order (Figures 4B,C).

In our dataset, we observed that the prediction of nCRT responders had a sensitivity and specificity of 83.33% (10/12) and 61.90% (13/21), respectively, with an accuracy of 69.70% (95% CI, 64.53–74.87%). The AUC was 0.800 (95% CI: 0.647–0.952; Figure 4D).

### The 41-GPS Predicts Survival and Treatment Response in LARC and Colorectal Cancer

**Figure 5:**
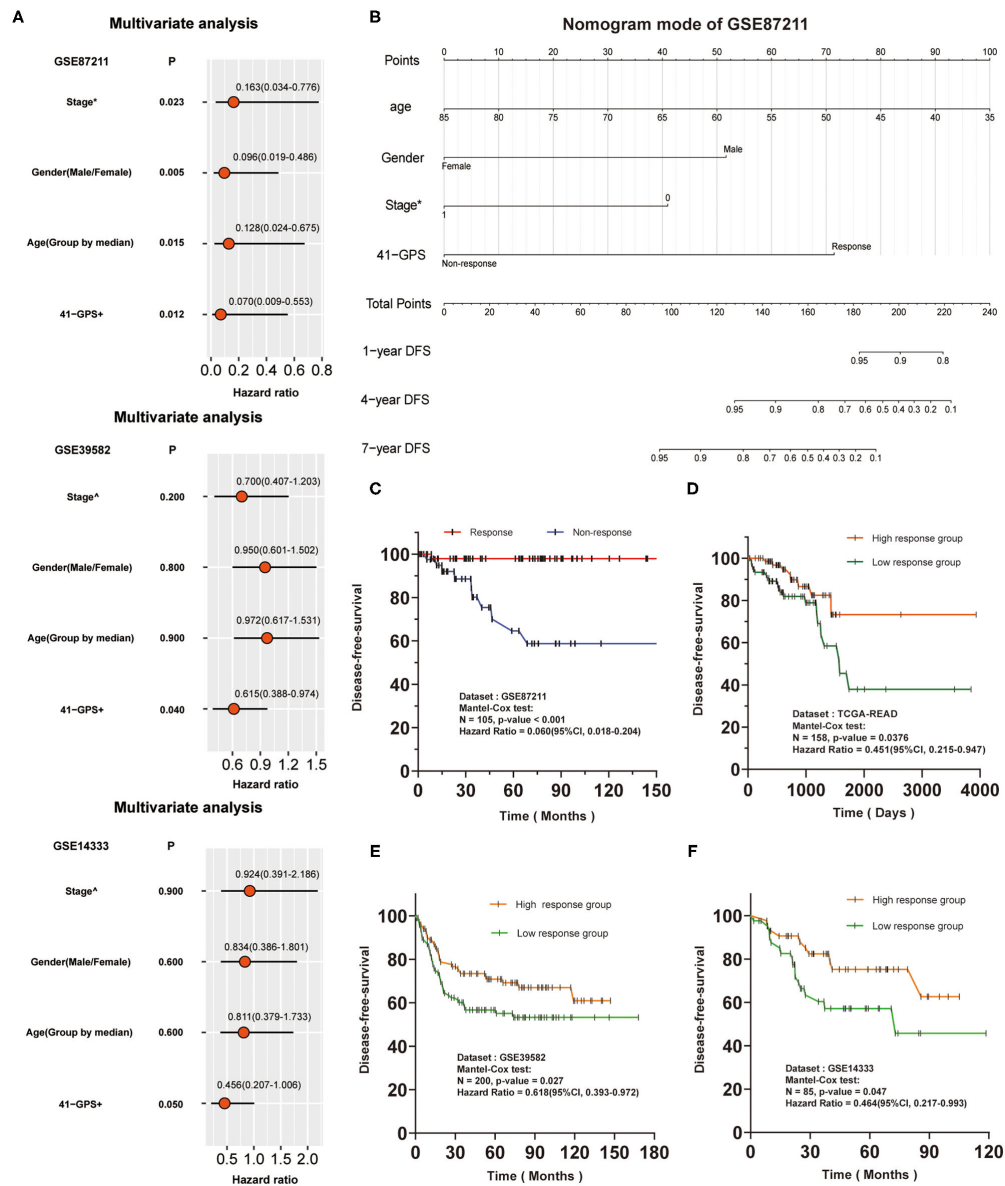
Independence verification and survival analysis verification of 41-GPS. Stage*: The status of lymph node metastasis before surgery, 0 means no metastasis, 1 means metastasis. Stage∧: The stage of colon cancer. 41-GPS+: In multivariate analysis of GSE87211, patients were divided into responders and non-responders. In multivariate analysis of GSE39582 and GSE14333, patients were divided into high-response groups and low-response groups according to the number of responding gene pairs. **(A)** Univariate analysis of GSE87211, GSE39582 and GSE14333. Hazard ratio and *P*-values were calculated using log-rank test. **(B)** Nomogram models of GSE87211. **(C)** Disease-free survival rate of 105 locally advanced rectal cancer patients in GSE87211. **(D)** Disease-free survival rate of 156 rectal cancer patients in TCGA-READ. **(E)** Disease-free survival rate of 200 colorectal cancer patients in GSE39582. **(F)** Disease-free survival rate of 85 colorectal cancer patients in GSE14333.

We conducted a survival analysis on the DFS data of 105 patients in the training cohort, and tested whether 41-GPS could predict the benefit of post-operative chemotherapy of LARC. Among the 105 samples, 58 were predicted to be responders and 47 non-responders. After adjusting for stage, age, and gender, the average DFS time of responders was 56.2 months, which was significantly longer than that of non-responders [38.3 months; Cox test, *p* < 0.001, hazard ratio (HR) = 0.060, 95% CI, 0.018–0.204; Figure 5C].

As there was no response-related information for GSE39582 and GSE14333, we divided the patients into high- and low-response groups based on the median of response genes in 41-GPS. Comparison of the HR values of all indicators revealed 41-GPS as the best predictor (Figure 5A). Multivariate analysis of GSE87211 and nomogram model showed that 41-GPS was an independent predictive indicator and did not overlap with other indicators (stage, gender, age), suggesting that 41-GPS may be an effective predictor of the pathological response of LARC patients (Figures 5A,B). In TCGA-READ analysis, we found that the DFS time of the high-response group was much higher than that of the low-response group (Figure 5D).

Additionally, we predicted the survival benefits of 285 patients with colorectal cancer. The genomic characteristics of colon and rectal cancers are very similar (30). Compared to those without pathological response, patients with pathological response can obtain the same advantages in prognostic survival benefits from post-operative chemotherapy (31). For this reason, we hypothesized that 41-GPS could reflect the authenticity of the prediction effectiveness in those datasets. Considering the differences between the cohorts, we divided the patients into high- and low-response groups by the median number of response gene pairs (a gene pair conforming to *G*_*a*_ > *G*_*b*_in 41-GPS, where *G*_*x*_ is the expression value). The results showed that the prognosis of patients in the high response group was considerably better than that in the low response group (Figures 5E,F).

### The 41-GPS Reflects the Drag Resistance of Cell Lines

**Figure 6:**
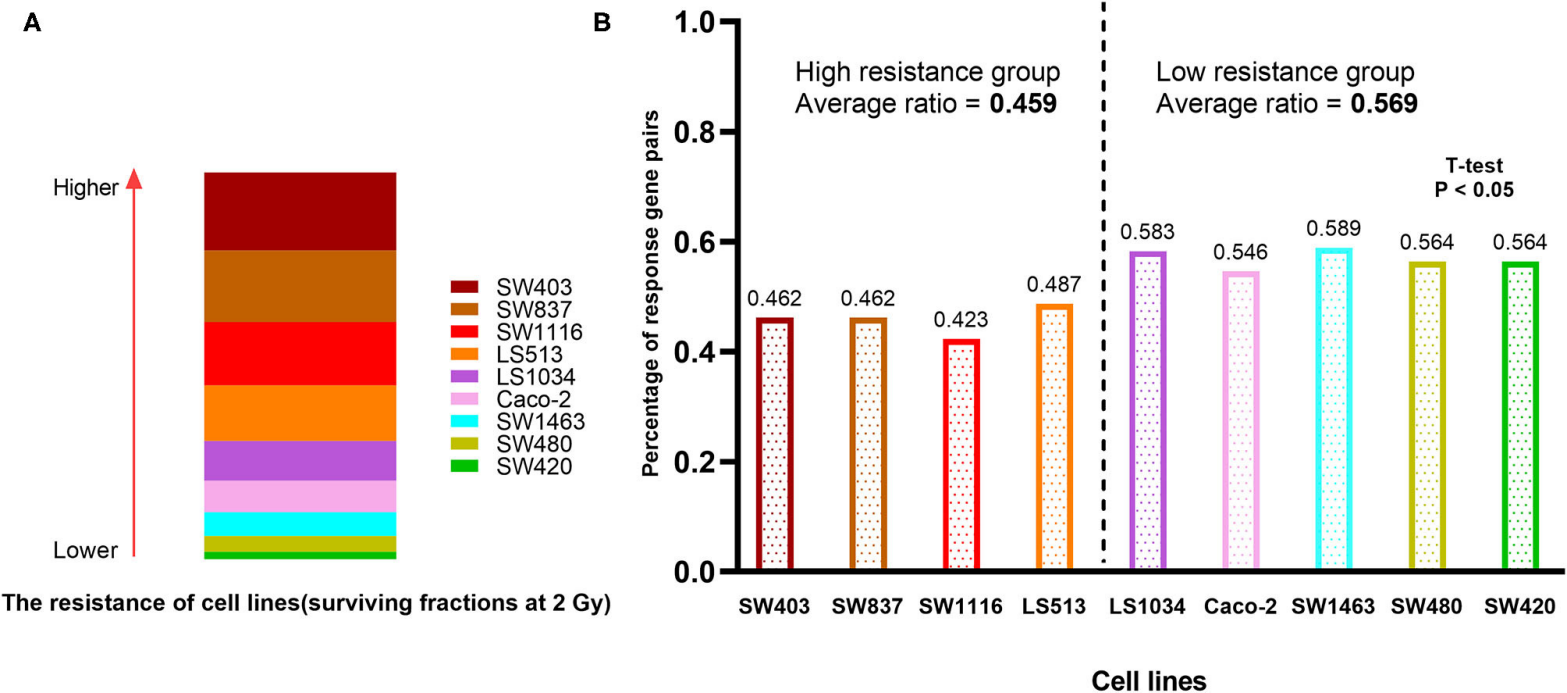
Cell line verification. **(A)** The resistance of nine colorectal cancer cell lines to chemoradiation. The rectangular area represents the resistance of the cell line to chemoradiation. **(B)** The percentage of response gene pairs in resistant and sensitive group.

We compared the chemoradiation resistance of nine colorectal cancer cell lines, of which SW403 and SW837 were the most resistant (Figure 6A). To test whether 41-GPS prediction would reflect the resistance of cell lines, we divided the patients again into sensitive and resistant groups based on the median resistance to chemoradiation and compared the number of response gene pairs between the two groups. We then used the 41-GPS to predict each cell line and calculated the proportion of response gene pairs. The results showed that the average response ratio of the resistant group was 0.459, which was lower than that of the sensitive group (0.569; *T*-test, *p* < 0.05; Figure 6B). These findings confirm the validity of our signature.

## Discussion

The identification of transcriptional predictive signatures of LARC response to nCRT would provide an additional reference for selecting patients most likely to benefit from therapy. In this study, we identified a predictive signature for the LARC response to nCRT based on REO and random forest algorithms. In previous studies, the REO algorithm showed resistance to experimental batch effects (7). Based on this, a more efficient and robust strategy (random forest) was used to identify our signature, which solved the limitations of our previous method regarding the selection of starting features (17). This signature performed well in predicting the pathological response and long-term survival prognosis of LARC patients receiving nCRT, and its prediction effect was also highly correlated with the resistance of colorectal cancer cell lines. Our experimental results showed the superiority of the random forest algorithm in omics data and the robustness of the 41-GPS. We believe that the 41-GPS could strongly predict nCRT responders prior to treatment. It is worth mentioning that cohort GSE87211 in our study contained both classification labels and survival data. This is direct evidence of the effectiveness of the 41-GPS. To the best of our knowledge, this is the first study to apply such data to the verification of rectal cancer markers.

This study, however, is subject to limitations. When the measurement scale or the number of categories of the predictor variables are different, the results of the random forest will be biased; however, this is inevitable (32). In the near future, more robust random forest models should be explored in large cohorts to define the importance metric influencing accuracy.

In conclusion, this study identified a signature of 41 gene pairs that can predict the nCRT response of LARC. This signature may be useful for individual clinical applications. It may help clinicians avoid risks for patients who will not benefit from 5-FU-based nCRT therapy.

## Data Availability Statement

The datasets presented in this study can be found in online repositories. The names of the repository/repositories and accession number(s) can be found in the article/Supplementary Material.

## Author Contributions

ZX and YG: conceptualization. YG: funding acquisition and project administration. ZX, SY, and YG: methodology. YL, MH, YD, LL, and WP: supervision. ZX: writing—original draft. ZX, SY, HC, GH, and YG: writing—review and editing. All authors contributed to the article and approved the submitted version.

## Funding

This study was supported by National Natural Science Foundation of China (NSFC, 82060618), Key Research and Development Program of Jiangxi Province (20203BBGL73184), Doctoral Fund of First Affiliated Hospital of Gannan Medical University.

## Conflict of Interest

The authors declare that the research was conducted in the absence of any commercial or financial relationships that could be construed as a potential conflict of interest.

## Publisher's Note

All claims expressed in this article are solely those of the authors and do not necessarily represent those of their affiliated organizations, or those of the publisher, the editors and the reviewers. Any product that may be evaluated in this article, or claim that may be made by its manufacturer, is not guaranteed or endorsed by the publisher.
